# Evaluating the Performance Characteristics of Pressure Monitoring Systems

**DOI:** 10.3390/s25020398

**Published:** 2025-01-11

**Authors:** Silvia Caggiari, Liudi Jiang, Peter Worsley

**Affiliations:** 1 Skin Sensing Research Group, School of Health Sciences, Faculty of Environmental and Life Sciences, University of Southampton, Southampton SO17 1HE, UK; p.r.worsley@soton.ac.uk; 2 School of Engineering, Faculty of Engineering and Physical Sciences, University of Southampton, Southampton SO17 1BJ, UK; l.jiang@soton.ac.uk

**Keywords:** interface pressure, pressure monitoring systems, mechanical test methods, performance characteristics

## Abstract

Measuring interface pressure is currently used in a variety of settings, e.g., automotive or clinical, to evaluate pressure distribution at support surface interfaces. Commercial pressure sensing arrays are employed to monitor and visualise these pressure distributions to aid mattress or cushion selection by assessing their ability to redistribute the pressure magnitudes over vulnerable areas, e.g., the buttock. These technologies vary in configurations and measurement principles, with manufacturers supplying calibration and specifications. This study evaluated the performance of six commercial pressure sensing arrays, which differed in sensor type, configuration, and spatial resolution. Each system was subjected to mechanical compression loading on a standard cushion using a dual hemispherical ‘buttock shaped’ standard indenter. Pressure parameters were estimated, e.g., contact area, peak pressure index, from the sensing arrays and a comparison between measured and predicted pressure values was performed. The results showed that both contact area and pressures are influenced by the spatial resolution, with higher values associated with systems with the highest resolution. A high variability between systems was observed in the measured pressure, with sensor type driving difference between the observed and the predicted pressures. Further research is needed to establish standards and performance analysis of these technologies.

## 1. Introduction

Over the last decades, interface pressure measurements have been employed in the automotive setting to assess the conditions at the seat interface. Their use has also become common in clinical settings to monitor the distribution of pressures at the interface between an individual and the underlying mattress or cushions whilst lying or sitting. These technologies are used as an aid for the prevention and treatment of pressure ulcers (PUs) [1].

Commercial systems typically involve high-resolution pressure-sensing arrays and are employed to evaluate the performance of both support surfaces and repositioning strategies. They provide a coloured contour map, which depicts the magnitude and area in which pressures are spatially distributed, providing biofeedback to both patients and healthcare professionals. These technologies employ pressure sensors which adopt a range of different measurement principles, e.g., capacitive, resistive, pneumatic, each with different operating performances. In a historical review, the performance of resistive (Tekscan system, Tekscan Inc., South Boston, USA) and pneumatic sensing arrays (Talley Pressure Monitor 3, TPM3, Talley Medical, Romsey, UK) have been compared, with the latter providing more accurate, stable, and reproducible pressure values, but were limited in terms of acquisition speed (1 sample every 60 s) and data presentation [2]. Commercial companies have developed different configurations designed to measure pressure distributions under individuals in beds [3], wheelchairs, or leisure chairs [4], which involve differing sensing area, spatial, and temporal resolutions. This inevitably influences the relative accuracy and repeatability of the pressure measures. In addition, system manufacturers provide calibration and specifications, defining sensitivity and accuracy, although standards for these features need to be established. Indeed, a “gold standard” sensor system has yet to be defined [5].

To provide information on whether pressure monitoring technologies could fit into clinical assessments, the International Organisation of Standardisation (ISO) developed a technical report (ISO/TR 16840-9:2015(E)) [6] to guide users in the performance of the tasks directly involved in their clinical use. However, this only includes definitions and interpretation of data, and it is limited to seating sensing arrays. To date, technical reports that describe test methods to assess the performance characteristics of pressure monitoring systems have yet to be established.

Bench test methods have been documented to assess the performance of cushion technologies. These involve the use of a dual semispherical indenter to characterise immersion and envelopment properties (ISO/FDIS 16840-12:2021(E)) [7]. Individual pressure sensors are used to instrument the indenter interface, and the pressure values are used to calculate envelopment characteristics. However, this only provides limited insights on the cushion envelopment and do not reflect typical clinical situations where pressure monitoring systems are used to assess the performance of a support surface.

An early study of interest investigated the influence of four different interface pressure mats on envelopment, immersion, and pressure distribution at the seat–cushion interface of seven cushions, using two buttock models (rigid and gel) and a static load of 500 N [8]. The authors observed that the presence of the sensor mats influenced the interface pressure magnitude and immersion, with some folding and/or bending observed, which resulted in a change in the contact area. Differences in pressure values and immersion were also observed between buttock models. Currently, there are no standards to define the use of a rigid or gel buttock model.

We identified a knowledge gap, where test methods to characterise the performance of pressure monitoring systems has yet to be established. Therefore, the present study aimed to evaluate a range of pressure monitoring systems characterised by different configurations and spatial resolutions, when subjected to a ramp of static loading conditions on a standard cushion.

The objectives were as follows:

i. Apply a compression ramp.
ii. Evaluate pressure features such as contact area, peak pressure index (PPI), and average pressure which might be influenced by the spatial resolution.
iii. Compare the pressure parameters observed in the different interface pressure monitors against predicted values from the hemisphere indenter.

By corroborating sensor array performance characteristics through a standard bench test method, this study will provide key metrics which can be considered when implementing this technology in clinical settings.

## 2. Materials and Methods

Standardised mechanical tests were carried out in the purpose-built research laboratory (Testing and Structure Research Laboratory) at the Boulderwood campus (University of Southampton) with a servohydraulic testing machine (8802 Instron Ltd., High Wycombe, UK) set in load control, through which different ramp conditions were applied. The servohydraulic actuator had a force capacity up to ±250 kN and a 150 mm of stroke.

Six pressure monitoring systems from different commercial companies were tested. These included 3 bed and wheelchair mats, characterised by different spatial resolutions (Table 1). These were placed on top of a foam castellated cushion (Integrity, Sumed, Glossop, UK), with a surface area of 0.19 m^2^ and thickness of 0.83 m.

**Figure 1:**
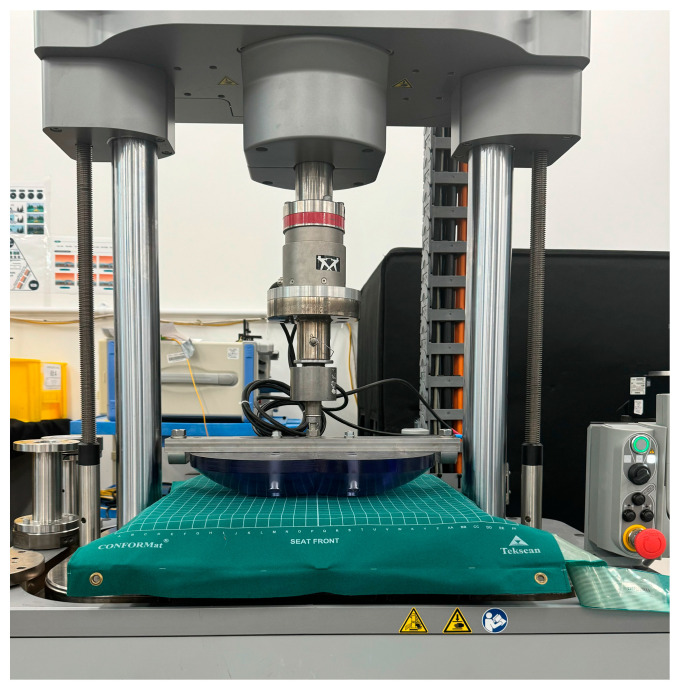
Testing setup with the ‘buttock shaped’ indenter and a pressure monitoring system placed on top of the cushion.

To perform the loading conditions, a ‘buttock shaped’ indenter characterised by two semispherical halves sectioned from a 380 mm diameter sphere was used (Figure 1). Each hemisphere was 3D printed in Onyx material, which is a micro carbon fibre filled nylon, using a Markforged MKII 3D printer (Markforged, Waltham, MA, USA). This represents a sufficiently rigid material, whose mechanical properties are reported in Table 2, that does not deform following the application of the loading conditions. It was constructed following the ISO/FDIS 16840-12:2021(E) [7] for the envelopment and immersion characterisation of seat cushions using a dual semispherical indenter.

Figure 1 shows the setup of the indenter attached to the loading machine, with the cushion and a sitting mat on top.

### 2.1. Testing Protocol

Prior to commencing the testing sessions, each pressure monitoring system was positioned on top of the cushion, which was placed under the indenter, with the latter positioned at 125 ± 25 mm from the rear of the cushion.

Starting from 25 N, twelve loading conditions were applied, while force data were continuously recorded via proprietary commercial software (Bluehill^®^ Universal, Instron Ltd., High Wycombe, UK). The loading conditions involved 25 N, 50 N, and 100 N after which increments of 100 N up to 800 N were applied, each held for 5 min. The interface pressure distributions were continuously recorded throughout the test period. This provided corresponding pressure values between 10 mmHg and 60 mmHg which are typically observed in lying and sitting postures [9,10,11].

The load was then removed to allow the cushion to recover for a minimum of 5 min prior to the next test.

### 2.2. Data Analysis

From the continuous pressure distribution, parameters such as the contact area [cm^2^] between the indenter and each of the pressure mats, peak pressure index (PPI) defined as the mean of the 10 highest pressure values, and the average pressure [mmHg] were estimated for each captured frame using a custom written Matlab script (Matlab R2024, Mathworks, Natick, MA, USA).

Signals were resampled at a frequency of 1 Hz and filtered using a moving average filter with a window length of 10 samples to remove noise.

To briefly review, for each captured frame, the contact area describes the number of sensors recording a pressure of or above a minimum operating range threshold, PPI describes the mean of the 10 highest pressure values, and the average pressure describes the mean of the pressure distribution. These features are commonly used as clinical indicators for the efficacy of cushion and/or mattress selection and postural correction in patients at risk of pressure ulcers.

Data from the compressive loading ramp [N] were also collated from the loading device and used to estimate the predicted pressure values. This involved dividing the measured load by the estimated contact area (measured pressure values > 5 mmHg) [N/cm^2^], reported in mmHg, as indicated by the formula below.

$$predicted\;pressure\;\left[mmHg\right]=\frac{Load\;[N]}{Contact\;area\;[{cm}^{2}]}$$

These predicted values were then compared with the average pressure estimated from each system.

## 3. Results

**Figure 2:**
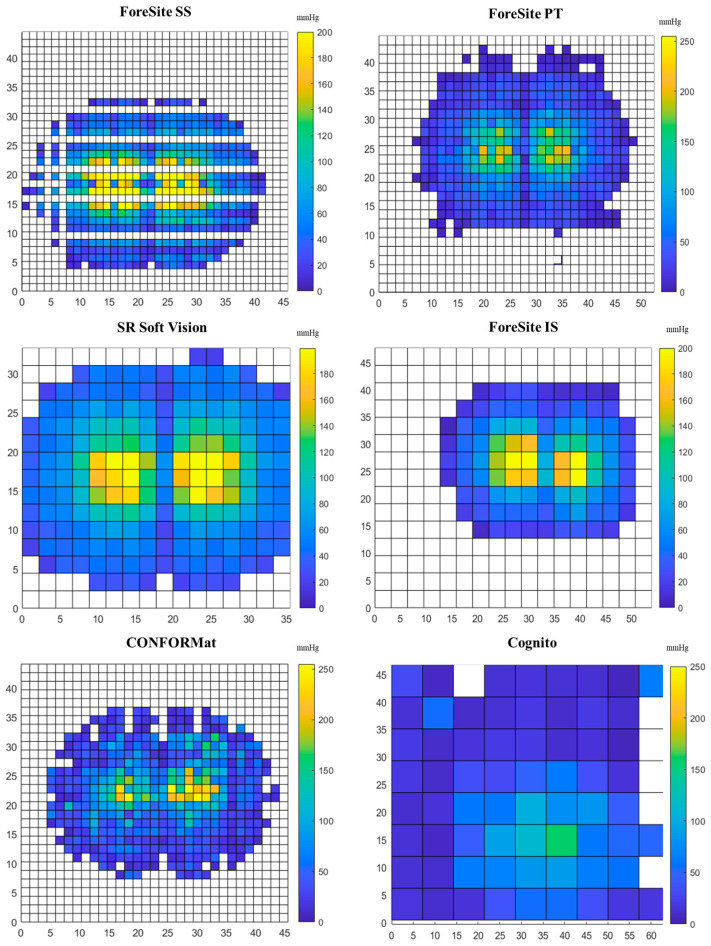
Snapshot of the pressure distribution at a loading condition of 800 N for all the pressure mats.

Figure 2 shows a snapshot of the distribution of the pressures recorded when a force of 800 N was applied to each pressure mat. It is immediately notable that the contour of the pressure distribution differs at the different spatial resolutions, with the colour gradient depending on the number of sensors within the acquisition area for system.

As depicted in Figure 2, it was interesting to observe that the ForeSite SS, which is characterised by highest spatial resolution (1.27 cm), showed empty pressure values at the area of contact with the indenter. This could be attributed to both the mat’s resolution and creases at the cushion interface. By contrast, the Cognito system, which is characterised by the lowest spatial resolution (>6.3 cm), was affected by a high background noise that cannot distinguish the contour of the indenter, affecting the estimation of the contact area.

The results showed that contact area is affected by the spatial resolution. Table 3 summarizes the median values (±interquartile range (IQR)) at each loading condition. The Cognito system recorded the highest contact area at all loading conditions (>3000 cm^2^), with these values influenced by the background noise. For this reason, it will be excluded from the subsequent description of the results.

Analysis of the data highlighted that the ForeSite SS and PT, which are the sitting and lying mats with the highest resolution of 1.27 cm and 1.59 cm, respectively, recorded the highest contact area values. ForeSite IS did not record any pressure values at both 25 N and 50 N, due to the proprietary algorithm which triggers the pressure sensels when their number and pressure values exceed a specific threshold. By contrast, VR Soft Vision system with its spatial resolution of 2.2 cm revealed lower values with this particularly exacerbated when low forces were applied, e.g., 21.7 (±0.5) cm^2^ at 25 N.

Closer examination of the data highlighted that all pressure mats showed an approximately similar magnitude of change in contact area between loading ramp conditions, with the highest values recorded when the load increased from 100 N to 200 N (>40%) and the smallest values recorded for loads > 600 N (<6%). In addition, at the same loading condition, the difference in contact area between mats was greatest for low forces, with this difference gradually decreasing to values below 10% for loading conditions ≥ 300 N.

Table 4 summarizes the median values (±IQR) of the PPI at each loading condition. All systems showed an increase in the PPI values associated with an increase in load, but this does not follow a consistent trend.

Data associated with the seating mats (ForeSite SS, Soft Vision, CONFORmat) showed that the higher the resolution the higher the PPI, with the ForeSite SS showing a median value equal to the maximum pressure allowed of 255 mmHg, when loaded at 800 N. Also, the VR Soft Vision and CONFORMat systems showed some pressure sensels that saturated at the maximum recording range of 199.5 and 255 mmHg, respectively (Figure 2). This is also evident in the ForeSite IS. By contrast, none of the pressure sensels of the ForeSite PT recorded a pressure of or above 250 mmHg (Figure 2). Comparison between ForeSite PT and IS highlighted that the higher resolution system (PT) recorded greater PPI values at each loading condition. By contrast, the Cognito system showed approximately similar PPI values (<15% difference) to the ForeSite PT at low loading conditions, e.g., 25 N to 400 N, with these consistently increasing from 25% to 50% during the subsequent loading conditions.

**Figure 3:**
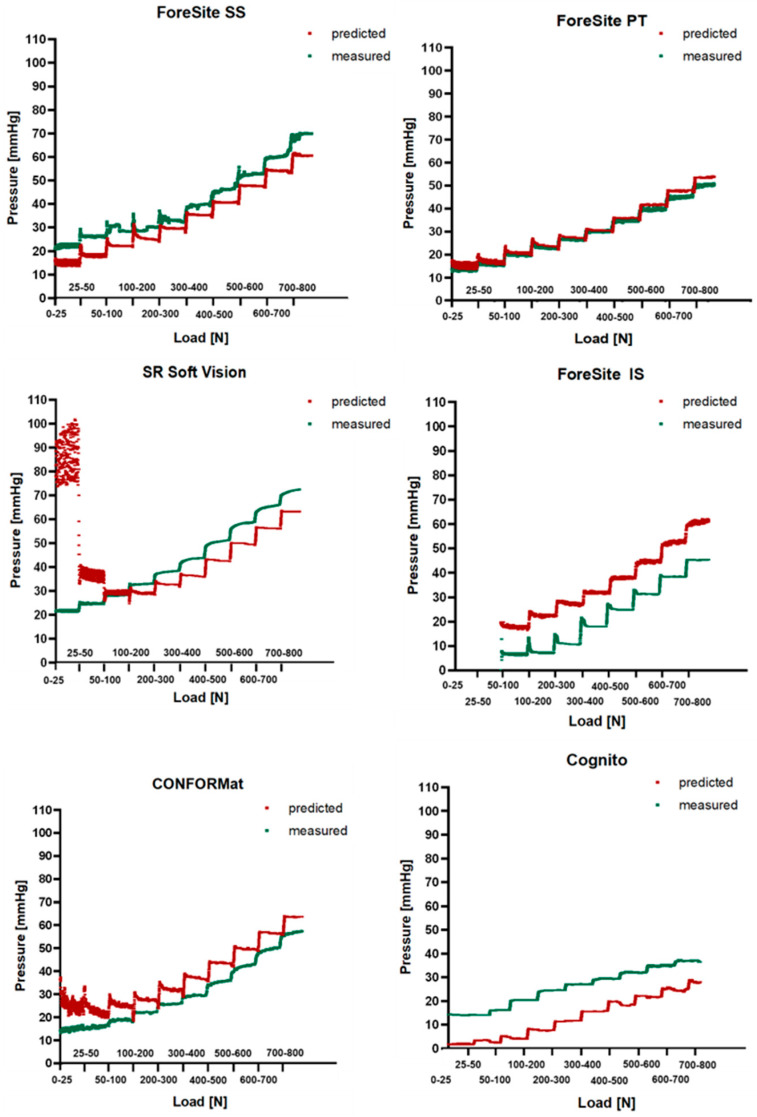
Comparison between predicted (in red) and measured (in green) pressure values at all loading conditions for all systems.

Comparison between predicted and measured pressure is shown in Figure 3 for all systems. These figures revealed that at low loading conditions e.g., <300 N, the predicted pressure signals showed a high signal to noise ratio, which was particularly exacerbated in the SR Soft Vision system. Here, the small contact area generated high predicted pressure values.

Table 5 summerises the mean difference (±standard deviation (SD)) between the predicted and the measure pressures at each of the loading conditions. These values were measured during approximately the last 3 min of each loading condition. Analysis of the data showed that the ForeSite PT displayed approximately similar predicted and measured pressures, with the difference between these values ranging between 0.5 and 3.5 mmHg, and higher differences associated to both lower and higher loads e.g., 25 N and >500 N, respectively. By contrast, the Soft Vision system showed a high degree of error in the predicted pressure, which is particularly evident at 25 N. This resulted from the small measured contact area (Table 4).

The differences between predicted and measured pressures did not follow a consistent trend in relation with both the resolution of the systems and the loading conditions. However, it is worth of noting that all systems with the exception of the ForeSite SS and Cognito, showed higher predicted pressure values, as indicated by the positive mean difference in Table 3. In addition, the majority of the systems, showed the lowest difference between predicted and measure at low loading conditions, e.g., 100 N and 200 N. 

## 4. Discussion

The present study aimed at evaluating the performance of six pressure monitoring systems characterised by different principles, e.g., capacitive, resistive, configurations, and spatial resolutions (Table 1), when subjected to a ramp of static loading conditions on a soft foam cushion. The test method proposed in the study involved ramped loading conditions, each maintained for a period of 5 min, with the data from both the Instron device and pressure sensing arrays revealing some hysteresis related to the viscoelastic properties of the foam cushion, which were resolved after approximately 1 min of each ramped load (Figure 3).

The results demonstrated that pressure features such as contact area and PPI were affected by the spatial resolution (Figure 2), with the highest values associated with systems with the greatest spatial resolution (Table 3 and Table 4). Comparison between predicted and measured pressures showed approximately similar values for the ForeSite PT, but some deviations in other systems which differed between sensing arrays (Figure 3, Table 5). In some cases, e.g., ForeSite SS and Cognito, the measured values were higher than the predicted as opposed to the ForeSite IS which showed an inverse trend. Deviation between measured and predicted values could be influenced by the contact area, whose values differed between the different configurations (Table 3).

This work highlights the heterogeneity of commercial systems present in the market. These systems are typically used in clinical practice to evaluate the performance of support surfaces and assist in their selection, with the manufactures supplying calibration and specifications, and defining sensitivity and accuracy. This study highlights the need for more standardization and a set of performance metrics by which these systems can be assessed against. Indeed, the current ISO standards (ISO/TR 16840-9:2015(E)) [6] do not include test methods on pressure monitoring systems to evaluate their performance, and, to date, no data of sensitivity and accuracy of the pressure measurements are available. Thus, to the authors’ knowledge, this is the first study exploring the performance characteristics of different pressure monitoring systems subjected to a series of loading increments.

Previous research has implemented mechanical testing methods to evaluate the performance of different cushions [12]. As an example, a study of interest [8] assessed seven cushions characterised by different designs and materials applying a static load of 500 N with two ‘buttock shaped’ indenters, rigid and gel, which were instrumented with five individual pressure sensors. Four different pressure monitoring systems with different principles, e.g., capacitive and resistive, were used to investigate whether the presence of a mat at the buttock–cushion interface influenced the pressure magnitude, immersion, and envelopment at the specific locations. Direct comparison with our study is difficult as they investigated the pressure magnitude from the five pressure sensor locations; however, the authors highlighted a large variability in the measurements which depended on the resolution and type of mat. Another early study of interest also reported that pressure mats do not provide highly accurate and repeatable readings [13]. A Force Sensitive application (Vista Medical, Winnipeg, Minnesota) mat was tested following the ISO N 338, 2001 [13]. This was placed on top of 10 different cushions which varied in material construction and contouring and subjected to three repeated loads of 500 N, applied with a gel Skeletal Embedded Loading indenter. The results showed a high variability of the total force [N], calculated as the sum of the pressure readings multiplied by the sensing area, with a poor intra-class correlation coefficient (ICC). By contrast, parameters such as average pressure, peak pressure index, and contact area were found reliable. Although Sprigle et al. [13] used a different indenter, the measured average pressure of ~30 mmHg on a foam cushion was observed when subjected to a load of 500 N. These values are comparable to the present study in similar loading conditions when using the ForeSite PT and IS, Cognito, and CONFORMat systems. The remaining systems measured a higher pressure of >40 mmHg.

The present study has some limitations. We used a dual hemispherical buttock shaped indenter and a relatively soft foam cushion, typically used in clinical practice, which limit the generalizability of the results. The material properties of the cushion, e.g., viscoelastic effect, might have influenced the pressure readings, particularly at lower load conditions. Therefore, we acknowledge that further investigation is needed across different support cushions. In addition, albeit ISO standards currently use a rigid model, previous studies have used a gel buttock model. Thus, there is the need to establish standard test methods and assess the pressure outcomes combining different indenters and support surfaces. Our study involved a single assessment of each system, therefore future studies could investigate the repeatability of the measurements.

This study implemented a bench test method involving mechanical compression loading to investigate the performance characteristics of six pressure sensing arrays. Key pressure metrics such as contact area and PPI were used to compare predicted and observed pressure values. Our study represents the first of its kind in evaluating the temporal performance of pressure sensing technologies and may contribute to establishing standard test methods which could be considered when implementing these technologies in the clinical settings, which have now been adapted to monitor over prolonged periods. From a research perspective, it is evidenced that future work is needed to characterise relative performance of different sensing arrays and what factors may influence the accuracy of measurement; for example, the effects of support surface, e.g., foam, air cell, or gel materials, and the implications of prolonged monitoring on key performance indicators such as signal drift. This will enable researchers and clinicians, who use the technology to understand the performance characteristics of different sensing arrays, to support clinical or research evaluations. Future works could also include theoretical models and finite element simulations for comprehensive analysis and modelling to assess load distribution across different sensing arrays.

## 5. Conclusions

This study evaluated the performance of six commercial pressure-sensing arrays, which differed in sensor type, configuration, and spatial resolution through a mechanical bench test. We demonstrated that key pressure metrics, e.g., contact area and peak pressure index, were influenced by the system’s spatial resolution, with higher values associated with systems with the highest resolution. In addition, we observed a high variability in the measured pressure between systems, with sensor type driving differences between the observed and the predicted pressures. There is the need to establish standards and performance analysis of these technologies prior to and during their implementation to clinical practice.

## Figures and Tables

**Table 1:** Specification of different commercial pressure monitoring system.

Pressure Monitoring System	Type of Sensor	Configuration	Specifications
ForeSite SS (XSensor, Calgary, AB, Canada)	Capacitive	Sitting	Spatial resolution: 1296 sensors (36 × 36 array)
			Sensing area: 457 × 457 mm^2^
			Sampling frequency: 5 Hz
			Operating range: 1.4–26.7 kPa (10–200 mmHg)
			Thickness: 0.1 mm
CONFORMat (Tekscan, Boston, MA, USA)	Resistive	Sitting	Spatial resolution: 1024 sensors (32 × 32 array)
			Sensing area: 471.4 × 471.4 mm^2^
			Sampling frequency: 1–8 Hz
			Operating range: 1–34 kPa (7.5–255 mmHg)
			Thickness: 0.134 mm
SR Soft Vision (Sumitomo Riko, Aichi, Japan)	Capacitive	Sitting	Spatial resolution: 256 sensors (16 × 16 array)
			Sensing area: 355 × 355 mm^2^
			Sampling frequency: 5 Hz
			Operating range: 1–266 hPa (0.75–199.5 mmHg)
			Thickness: 0.2 mm
ForeSite PT (XSensor, Calgary, AB, Canada)	Capacitive	Full body	Spatial resolution: 5664 sensors (118 × 48 array)
			Sensing area: 1880 × 762 mm^2^
			Sampling frequency: 1 Hz
			Operating range: 0.6–33 kPa (5–255 mmHg)
			Thickness: 0.1 mm
ForeSite IS (XSensor, Calgary, AB, Canada)	Capacitive	Full body	Spatial resolution: 1440 sensors (60 × 24 array)
			Sensing area: 1880 × 762 mm^2^
			Sampling frequency: 3 Hz
			Operating range: 1.4–26.7 kPa (10–200 mmHg)
			Thickness: 0.1 mm
Cognito Health (Boulder, CO, USA)	Resistive	Full body	Spatial resolution: 240 sensors—3 × (8 × 10 array)
			Sensing area: 1506 × 715 mm^2^
			Sampling frequency: 10 Hz
			Operating range: 0.6–33 kPa (5–250 mmHg)
			Thickness: 0.1 mm

**Table 2:** Tensile mechanical properties of Onyx (supplier values).

Mechanical Properties	Value
Young’s modulus *E* (MPa)	2400
Yield stress *Re* (MPa)	40
Tensile strain at break A (%)	25

**Table 3:** Contact area (median ± interquartile range) at each loading condition.

Contact Area [cm^2^]						
Force [N]	ForeSite SS	Soft Vision	CONFORMat	ForeSite PT	ForeSite IS	Cognito
25	125.8 (±0.7)	21.7 (±0.5)	76.0 (±7.0)	114.0 (±2.5)	/	3006.0 (±0.0)
50	207.7 (±1.6)	102.4 (±3.0)	167.0 (±13.8)	216.2 (±5.6)	/	3006.0 (±0.0)
100	338.4 (±0.6)	256.0 (±0)	299.1 (±12.5)	362.0 (±5.1)	280.2 (±5.0)	3006.0 (±0.0)
200	599.4 (±13.1)	516.9 (±3.4)	544.3 (±7.8)	627.5 (±12.9)	517.1 (±8.1)	3006.0 (±0.0)
300	759.7 (±5.0)	689.2 (±3.9)	702.5 (±13.9)	811.3 (±11.9)	699.6 (±20.2)	3006.0 (±0.0)
400	851.3 (±5.3)	822.6 (±8.4)	803.2 (±17.9)	977.6 (±7.8)	832.7 (±4.0)	3050.9 (±0.0)
500	923.7 (±2.1)	876.8 (±9.8)	858.7 (±8.3)	1040.3 (±3.0)	906.2 (±6.0)	3050.9 (±0.0)
600	942.7 (±3.1)	900.9 (±7.4)	904.1 (±9.1)	1071.9 (±4.2)	959.7 (±3.0)	3095.8 (±0.0)
700	969.2 (±3.7)	934.8 (±4.9)	927.0 (±8.0)	1087.8 (±5.1)	977.8 (±3.0)	3133.9 (±35.9)
800	991.4 (±4.8)	950.1 (±0)	943.4 (±2.6)	1102.5 (±3.5)	992.9 (±3.0)	3230.4 (±0.0)

**Table 4:** Peak pressure index (median ± interquartile range) at each loading condition.

PPI [mmHg]						
Force [N]	ForeSite SS	Soft Vision	CONFORMat	ForeSite PT	ForeSite IS	Cognito
25	37.0 (±0.1)	9.5 (±0.2)	22.1 (±1.3)	20.8 (±0.3)	/	24.9 (±0.9)
50	44.2 (±0.4)	27.0 (±0.0)	27.5 (±2.4)	26.1 (±0.4)	/	23.5 (±0.5)
100	54.9 (±2.4)	36.6 (±0.2)	41.1 (±1.6)	35.3 (±0.3)	25.1 (±0.3)	30.7 (±0.5)
200	62.3 (±0.2)	47.6 (±0.2)	62.3 (±1.7)	45.0 (±0.5)	34.7 (±0.2)	46.7 (±0.7)
300	77.9 (±0.8)	57.6 (±0.8)	82.0 (±2.0)	53.8 (±0.6)	43.5 (±0.2)	55.5 (±0.4)
400	102.5 (±0.8)	74.3 (±2.6)	98.4 (±2.0)	71.0 (±1.0)	56.0 (±0.5)	61.9 (±0.2)
500	139.9 (±3.1)	97.5 (±4.0)	117.5 (±6.3)	94.1 (±2.2)	77.5 (±1.6)	69.3 (±0.5)
600	178.4 (±3.1)	126.9 (±5.2)	151.7 (±5.8)	123.0 (±2.7)	106.1 (±2.6)	75.6 (±0.5)
700	227.1 (±3.8)	163.7 (±6.5)	195.7 (±8.9)	157.0 (±2.7)	143.9 (±3.4)	85.4 (±1.3)
800	255.0 (±1.1)	196.3 (±2.8)	237.8 (±1.9)	196.6 (±4.4)	186.4 (±4.3)	95.4 (±0.7)

**Table 5:** Mean difference (±SD) between the predicted and measured pressure at each loading condition for all systems. Negative values refer to higher measured values with respect to the predicted.

Force [N]	ForeSite SS	Soft Vision	CONFORMat	ForeSite PT	ForeSite IS	Cognito
25	−6.9 (±1.8)	64.9 (±8.0)	10.1 (±2.5)	2.0 (±1.5)	/	−10.4 (±0.3)
50	−7.9 (±1.3)	11.9 (±1.8)	6.6 (±1.8)	1.4 (±1.5)	/	−10.4 (±0.9)
100	−6.9 (±1.6)	1.2 (±0.7)	6.4 (±1.0)	0.9 (±0.9)	10.8 (±1.3)	−11.3 (±0.9)
200	−4.3 (±1.2)	−3.7 (±0.4)	5.3 (±0.8)	0.8 (±0.3)	14.1 (±2.4)	−12.4 (±0.9)
300	−3.5 (±0.8)	−5.3 (±0.3)	6.3 (±0.7)	0.9 (±0.4)	14.9 (±3.9)	−13.2 (±0.2)
400	−4.2 (±0.4)	−6.9 (±0.6)	7.9 (±0.7)	0.5 (±0.4)	12.3 (±3.2)	−11.5 (±0.1)
500	−5.2 (0.6)	−7.6 (±0.7)	8.3 (±0.8)	1.3 (±0.4)	11.8 (±2.7)	−9.9 (±0.6)
600	−4.8 (±0.5)	−8.2 (±0.8)	7.7 (±1.3)	2.0 (±0.4)	12.5 (±2.2)	−10.1 (±0.4)
700	−5.8 (±0.5)	−8.7 (±0.8)	7.2 (±0.9)	2.9 (±0.5)	13.8 (±1.4)	−9.7 (±0.5)
800	−8.2 (±1.4)	−8.5 (±0.6)	7.1 (±0.6)	3.5 (±0.4)	15.2 (±0.6)	−8.8 (±0.5)

## Data Availability

All data supporting this article will be made available by the authors on request.
